# Rapid In Vitro Pathological Diagnosis of Glioma Using Dual‐Color Quantum Dot Probes for Precise Intraoperative Resection

**DOI:** 10.1002/advs.76379

**Published:** 2026-07-06

**Authors:** Dongdong Wu, Chenxuan Yang, Yuanbin Wu, Minghang Liu, Yizhi Zhang, Yuang Cai, Yibo Zhang, Jimei Chi, Hengchao Ma, Ding Zhang, Chenqing Liu, Meng Su, Guochen Sun

**Affiliations:** ^1^ Department of Neurosurgery The First Medical Centre Chinese PLA General Hospital Beijing Beijing China; ^2^ Medical School of Chinese PLA Beijing China; ^3^ Department of Emergency Medicine, the Seventh Medical Center Chinese PLA General Hospital Beijing China; ^4^ Nankai University School of Medicine Nankai University Tianjin China; ^5^ Key Laboratory of Green Printing, Institute of Chemistry, Chinese Academy of Sciences (ICCAS), Beijing Engineering Research Center of Nanomaterials for Green Printing Technology Beijing National Laboratory for Molecular Sciences (BNLMS) Beijing China; ^6^ Department of Otorhinolaryngology, Head and Neck Surgery Sixth Medical Center of the Chinese PLA General Hospital Beijing China

**Keywords:** dual‐color probe, glioma, perovskite nanocrystal, precision medicine

## Abstract

Brain gliomas’ variable growth patterns and locations hinder complete surgical removal, while existing clinical imaging/histological methods fail to guide intraoperative resection. To address this, we developed blue‐green dual‐color probes (perovskite quantum dots conjugated to antibodies) targeting glioma cells and IDH1‐mutant simultaneously. These enable rapid intraoperative in vitro pathological diagnosis, helping surgeons devise resection strategies for personalized precise treatment. Tested on 56 clinical frozen sections, the combined detection using dual probes can be completed within 30 min with 91% accuracy: the glioma‐targeting probe identifies tumor boundaries, while the IDH1‐targeting one distinguishes subtypes for optimized margin delineation. We established a personalized resection strategy guided by these results; the dual‐target probes show great potential to assist neurosurgeons in precise glioma resection.

## Graphical abstract

Glioma resection surgery is challenging because of the infiltrative growth of the tumor into nearby tissue and the different surgical requirements for gliomas with different mutation types. To improve the precision and effectiveness of glioma resection, this study developed a new imaging probe that enables the tumor boundary and mutation status to be observed simultaneously during surgery.

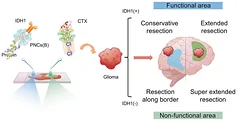

## 1 Introduction

Gliomas are among the most common malignant tumors of the central nervous system. Complete tumor resection is challenging because of the infiltrative growth characteristics of gliomas, leading to high overall recurrence and mortality rates [1, 2]. Surgery is the primary treatment for gliomas, and precise tumor resection is crucial for preventing recurrence and reducing complications [3]. To achieve effective resection treatments, the size of residual tumors must be minimized, the resection rate must be improved, and reasonable resection strategies with a strong scientific basis that are tailored to different molecular glioma subtypes must be formulated. Glioma subtypes with IDH1(isocitrate dehydrogenase 1) mutations grow slowly, are sensitive to radiotherapy and chemotherapy, and have a relatively favorable prognosis, whereas wild‐type IDH gliomas grow rapidly, are insensitive to radiotherapy and chemotherapy, and have a relatively poor prognosis [4, 5, 6, 7]. Therefore, the IDH1 mutation status is significantly correlated with the extent of surgical resection. Especially when tumors are adjacent to functional areas, to balance tumor resection and functional preservation, for IDH‐mutated gliomas, a limited resection strategy can be adopted for tumors in functional areas during surgery, with partial residual lesions retained. Subsequent sequential therapy combining radiotherapy and chemotherapy can eliminate residual tumors, thereby reducing the risk of functional area injury. However, conventional methods are unable to simultaneously achieve “boundary identification and molecular subtyping.” Existing genetic testing techniques also face challenges such as time‐consuming procedures, high technical complexity, and delayed results, which can easily lead to incomplete or excessive resection, making them unsuitable for intraoperative applications [8]. To achieve precise glioma resection, developing a rapid and efficient in vitro pathological detection technique to guide surgeons during surgery is urgently necessary.

Fluorescent probes can specifically bind to or react with target molecules or ions, which indicate the presence or concentration of the target through changes in fluorescence signals [9]. Among the diverse fluorescent materials, all‐inorganic lead‐halide (CsPbX3 (X = halide)) perovskite quantum dots (PQDs) exhibit excellent optoelectronic properties. As emerging fluorescent probes, the main advantage of PQDs is their high fluorescent quantum yield (>90%), which provides extremely strong fluorescence signals [10, 11, 12, 13]. By adjusting the halogen composition or nanocrystal particle size, the emission wavelength can be precisely controlled over a wide range (from ultraviolet to near‐infrared). In addition, the full width at half‐maximum (FWHM) of the emission peak is usually narrow (less than 20 nm), resulting in excellent spectral resolution and the ability to distinguish between multiple fluorescent signals [14, 15, 16]. Compared to traditional organic dyes, CsPbX3 is more resistant to photobleaching, making it suitable for long‐term imaging or monitoring. The synthesis of CsPbX3 nanocrystals is simple, and targeted functionalization can be achieved through surface modification. Thus, CsPbX3 is a promising luminescent label for imaging, biosensing, and clinical diagnosis [17, 18].

The intraoperative frozen section immunofluorescence technique is a rapid pathological detection method combining frozen sectioning with immunofluorescence staining. Through the specific binding of fluorescently labeled antibodies to tissue antigens, it enables visual localization of protein markers under confocal or fluorescent microscopy, meeting the need for immediate intraoperative in vitro pathological diagnosis. In this study, we developed a PQD‐based probe containing green quantum dots (PNCs(G)) and blue quantum dots (PNCs(B)), which can simultaneously identify glioma cells and IDH1 mutations to guide surgeons and help achieve precise tumor resection. The green CTX probe can target and recognize chloride channel proteins present on the membranes of glioma cells, thereby enabling the specific differentiation of glioma and non‐glioma cells. The blue IDH1 probe can specifically recognize glioma cells with IDH1 mutations at the cytoplasmic level, enabling the assessment of the IDH1 mutation status of the glioma. These probes are used for fluorescent staining of intraoperative frozen sections, thereby guiding surgeons to precisely resect tumors and formulate scientifically rational resection strategies during surgery. This allows for surgical treatment strategies to be individualized for different glioma types and locations (proximity to functional areas), thereby maximizing tumor removal, function preservation, and surgical outcomes.

## 2 Results

### 2.1 Validation of the Clinical Significance of IDH1 Mutation Status

Figure 1A intuitively presents the systematic differences between IDH1‐mutant and IDH1‐wild‐type gliomas in terms of pathological grades, clinical behaviors, and treatment responses. The left side represents the IDH1 wild‐type, which mainly consists of WHO grade IV glioblastoma (GBM). It is characterized by a high cellular proliferation index, ill‐defined tumor boundaries, and clinically manifests as rapid growth, short median overall survival, and high recurrence rate. In terms of treatment, it often requires maximal surgical resection and is insensitive to conventional chemotherapy and IDH inhibitors. The right side represents the IDH1‐mutant type, which mostly includes WHO grade II/III gliomas. It has a low cellular proliferation index and relatively well‐defined boundaries, with a slow clinical progression, long median overall survival, and low recurrence rate. In treatment, the function‐preserving resection strategy is more preferred, and such tumors exhibit high sensitivity to radiotherapy, chemotherapy, and IDH1 inhibitors.

**FIGURE 1:**
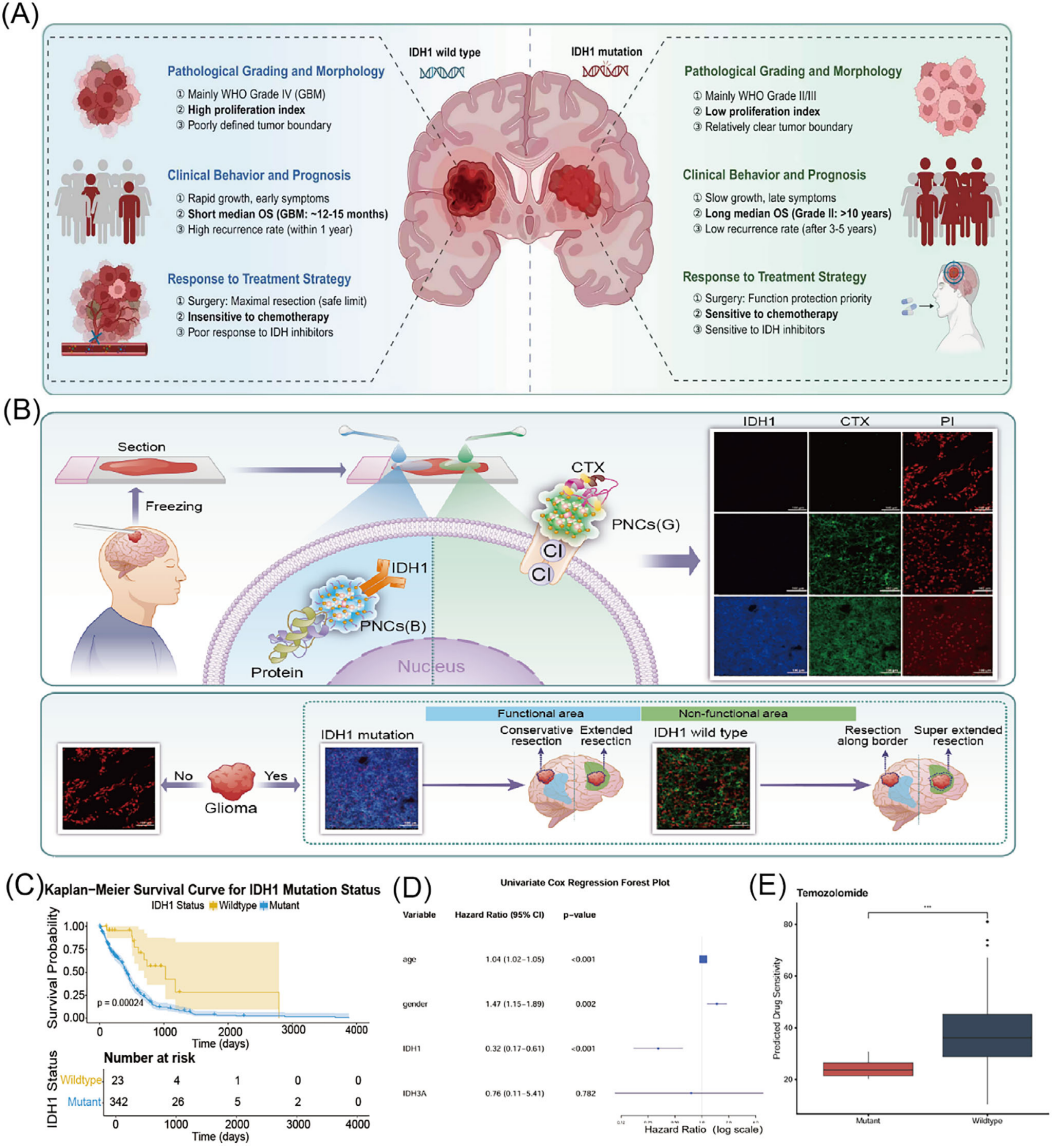
Clinical Significance of IDH1 Mutation Status in Gliomas and Application of Dual‐Color Quantum Dot Probes. (A) Schematic diagram showing the comparison of differences between IDH1‐mutant and IDH1‐wild‐type gliomas. (B) Schematic diagram of the application process and imaging of blue‐green dual‐color quantum dot probes targeting IDH1 and CTX in glioma tissue samples. (C) Bioinformatics analysis of Kaplan–Meier survival curves in glioma patients; (D) bioinformatics results of Cox regression analysis in glioma patients; (E) bioinformatics comparison of chemotherapeutic drug sensitivity.

Based on the aforementioned differences, this study further developed a dual‐color fluorescent probe applicable for rapid intraoperative ex vivo identification in pathological diagnosis of gliomas. As shown in Figure 1B, by replacing the original long‐chain oleylamine with the short‐chain ligand CBA, we successfully constructed blue‐green dual‐color perovskite nanocrystals with excellent water and oxygen stability. Furthermore, using charge coupling technology, we achieved stable conjugation of quantum dots with antibodies in aqueous solution, thereby developing dual‐color probes targeting two key targets: CTX and IDH1 mutation.

To further verify the clinical significance of IDH1 typing, we conducted an in‐depth analysis of The Cancer Genome Atlas (TCGA) and Chinese Glioma Genome Atlas (CGGA) databases. As shown in Figure 1C, patients with IDH1 mutation had significantly longer survival after radiotherapy (*p* = 0.00024). Cox regression analysis (Figure 1D) further confirmed that IDH1 mutation is an independent prognostic protective factor (HR = 0.32, 95% CI: 0.17–0.61, *p* < 0.001). Furthermore, the prediction of chemosensitivity using the OncoPredict algorithm (Figure 1E) revealed that the IC50 value of temozolomide in IDH1‐mutant type was significantly lower than that in wild‐type (*p* < 0.05), indicating higher chemosensitivity. Collectively, the above results indicate that rapid identification of IDH1 mutation status during surgery holds important clinical significance for guiding the formulation of surgical strategies and protecting patients' neurological functions.

### 2.2 Characterization of Dual‐Color Quantum Dot Probes

To realize the above detection goal, we first synthesized blue‐green dual‐color perovskite quantum dots as the core luminescent material. A comprehensive series of material characterizations were then carried out to verify their morphology, crystal structure, composition and stability.

The transmission electron microscopy (TEM) image of the supernatant containing PNCs(G) in Figure 2A shows quantum dots with a size of 11 ± 4 nm and a corresponding lattice spacing of 0.41 nm, consistent with the (100) crystal plane of CsPbBr3. The scanning electron microscopy (SEM) image of PNCs(G) in Figure 2B shows that the coated quantum dots (QDs) are mainly spherical, with particle sizes ranging from 40 to 70 nm and an average size of 56 nm. Figure 2C presents a TEM image of PNCs(B) with a lattice spacing of 0.31 nm, consistent with that of CsPb(Br/Cl)3. The coated PQDs are predominantly spherical with an average particle size of 58 nm (Figure 2D). This indicates that PLGA has a similar coating effect on QDs of different compositions, forming water‐ and O‐resistant spherical structures.

**FIGURE 2:**
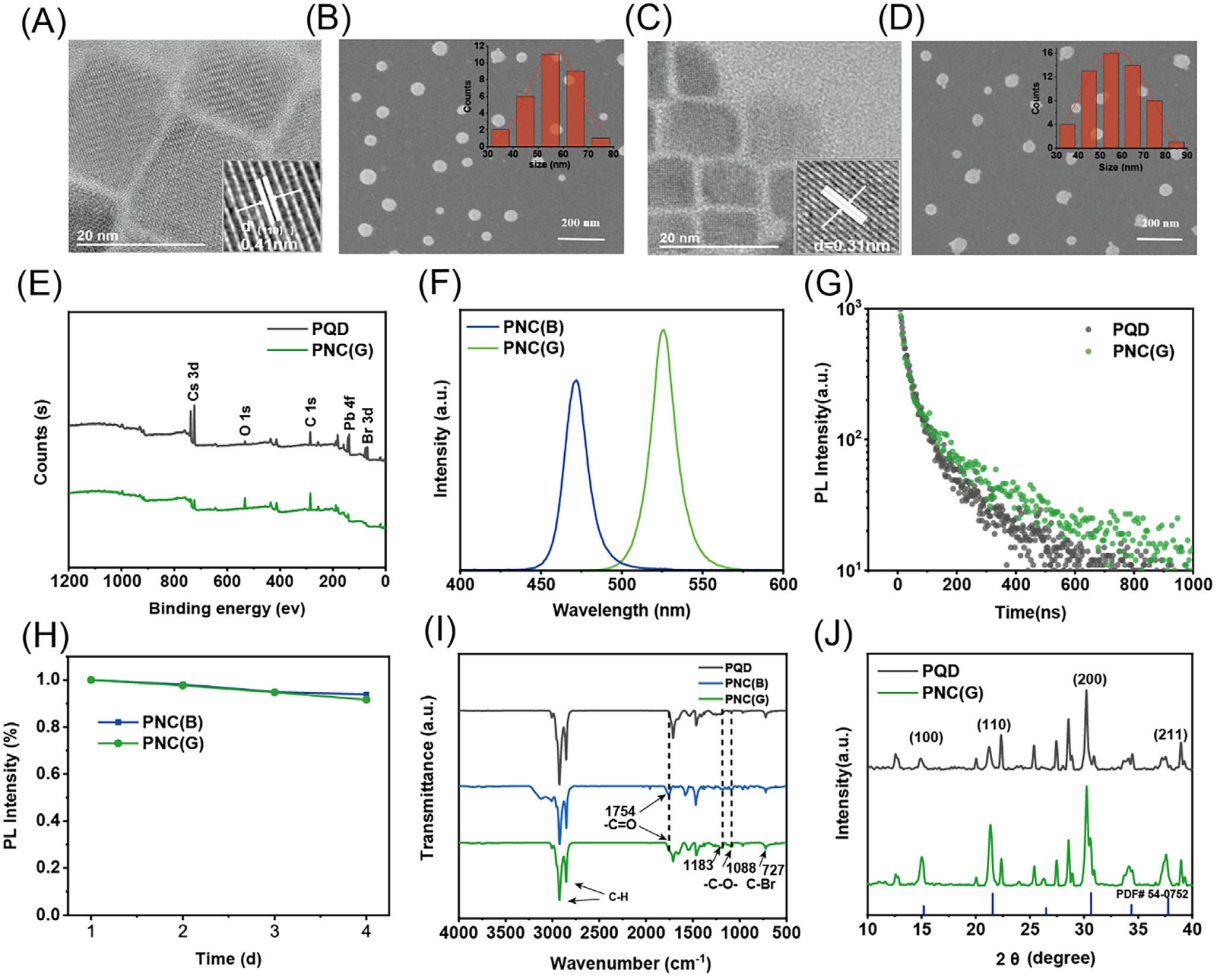
(A) TEM and (B) SEM images of PNCs(G); (C) TEM and (D) SEM images of PNCs(B); (E) XPS spectrum of PNCs(G); (F) PL spectra of PNCs(B) and PNCs(G); (G) TRPL spectra of PQDs and PNCs(G); (H) stability of the PL spectra of PNCs(B) and PNCs(G); (I) FTIR spectra of PNCs(B) and PNCs(G); and (J) XRD patterns of PQDs and PNCs(G).

To demonstrate that the PQDs were coated with the PLGA copolymer and bonded together to form the PNCs, an X‐ray photoelectron spectroscopy (XPS) analysis was conducted to confirm successful coating and ligand modification. The XPS spectra in Figure 2E imply the presence of Cs, Pb, Br, C, and O on the PQD surfaces. However, the surface of the P‐PNCs shows the presence of only C and O, because signals from Cs, Pb, and Br in the perovskite structure are masked by the PLGA coating. This indicates that the PLGA in the PNCs completely covers the PQDs, and the resulting structure is expected to enhance the stability of the PQDs significantly. Figure 2F shows the steady‐state photoluminescence (PL) spectra of PNCs(B) and PNCs(G). The emission peak of PNCs(B) is located at 472 nm with an FWHM of 17 nm, indicating blue emission. The emission peak of PNCs(G) is located at 526 nm with an FWHM of 19 nm, indicating green emission. These results confirm the successful preparation of blue and green PQDs. The time‐resolved PL (TRPL) spectra in Figure 2G show the results of a fitting analysis of the average PL lifetimes of the PQDs and PNCs(G). The fluorescence lifetime increases from 152.41 ns for the PQDs to 357.53 ns for PNCs(G). This is attributed to the strong interactions between the carboxyl groups in PLGA and the PQDs, resulting in a staggered network through polymer cross‐linking. This structure reduced the number of surface defects on the PQDs and enhanced the luminescence stability. As shown in Figure 2H, the PL fluorescence intensity of PNC(G) and PNC(B) was similar over 4 days of observation. This indicates that the PQDs coated with PLGA have good short‐term water and oxygen resistance.

The FTIR spectra of PQD, PNC(B), and PNC(G) are depicted in Figure 2I. The spectrum of PQD presents a relatively “simple” profile, with no prominent characteristic peaks at 1754 cm^−^^1^ (C═O stretching vibration), 727 cm^−^^1^ (C─Br vibration), or 1183 cm^−^^1^ (C─O stretching vibration), so it can serve as a “baseline spectrum” for comparing PNC derivatives. A strong absorption peak at 1754 cm^−^^1^ in the spectrum of PNC(B) corresponds to the stretching vibration of the C═O bond, confirming the introduction of carbonyl—containing functional groups, and there are differences in the functional group composition between PNC(B) and PNC(G). PQD can be regarded as the “basic structure” of PNC derivatives. Due to the differences in functional groups between PNC(B) and PNC(G), they may be products of different functionalization modifications of the same main chain or products resulting from differences in the synthesis routes. Figure 2J shows the XRD patterns of the synthesized PQDs and PNCs(G) used to confirm their crystal structures. The XRD patterns suggest that the PQD and P‐PNC samples contain two types of CsPbBr3 crystals (cubic and orthorhombic). The cubic phase can be identified by the peaks at 22.4°, 25.5°, 30.3°, 34.2°, and 37.6°, and the orthorhombic phase is identified by the peaks at 27.1° and 28.2°. Strong peaks are observed at 22.4°, 30.3°, and 37.6° for both the PQDs and PNCs, corresponding to the (110), (200), and (211) crystal planes of CsPbBr3 perovskite, respectively. Therefore, the main phase in the P‐PNCs remains unchanged after polymerization. This further proves that the successful coating of PQDs with PLGA copolymers can yield stable blue and green PQDs.

### 2.3 Preparation of Dual‐Color Quantum Dot Probes

Based on fully characterized PLGA‐coated perovskite quantum dots (PQDs) with excellent stability and luminescence properties, we further constructed a dual‐color targeted quantum dot probe by conjugating two specific antibodies, CTX and IDH1.R132H. As illustrated in the schematic diagram of Figure 3A, chlorotoxin (CTX) antibodies and isocitrate dehydrogenase 1 (IDH1) antibodies can be conjugated to aqueous solutions of green and blue perovskite quantum dots (PQDs), respectively, forming corresponding bioprobes. Specifically, the green probe targeting CTX can specifically bind to and recognize chloride channel proteins on the glioma cell membrane, thereby enabling the specific distinction between glioma cells and non‐glioma cells. The blue probe targeting IDH1 can specifically identify glioma cells carrying IDH1 mutations at the cytoplasmic level, thus allowing assessment of the IDH1 mutation status in glioma. The blue quantum dot probe (Figure 3B) exhibited distinct blue fluorescence upon excitation. Before conjugation with the IDH1 antibody, the blue quantum dot probe (PNC (B)) showed a high Zeta potential. After conjugation (PNC (B)‐IDH1), the potential significantly decreased (Figure 3C), confirming the successful modification of IDH1 antibodies onto the quantum dot surface and providing direct electrochemical evidence for the targeting capability of the probe. Photostability testing (Figure 3D) revealed that the photoluminescence intensity of the blue quantum dot probe only exhibited minimal attenuation over 30 days, demonstrating excellent photostability. This characteristic meets the requirements for signal stability in long‐term experiments or clinical sample detection, thereby reducing signal loss due to fluorescence decay. The green quantum dot probe (Figure 3E) displayed green fluorescence, and after conjugation with CTX (PNC (G)‐CTX), the Zeta potential also decreased markedly (Figure 3F). This result confirms the successful modification of CTX onto the quantum dot surface, providing direct electrochemical evidence for the probe's targeting ability. For the chromaticity coordinate analysis (Figure 3G), the CIE 1931 chromaticity coordinate diagram showed that the chromaticity coordinates of green quantum dots (PNCs (G)) and blue quantum dots (PNCs (B))—with PNCs (B) at (0.125, 0.089) and PNCs (G) at (0.146, 0.784)—belong to different regions. They exhibit high color discrimination, which can effectively avoid fluorescence crosstalk during dual‐color imaging and ensure the accuracy of imaging results.

**FIGURE 3:**
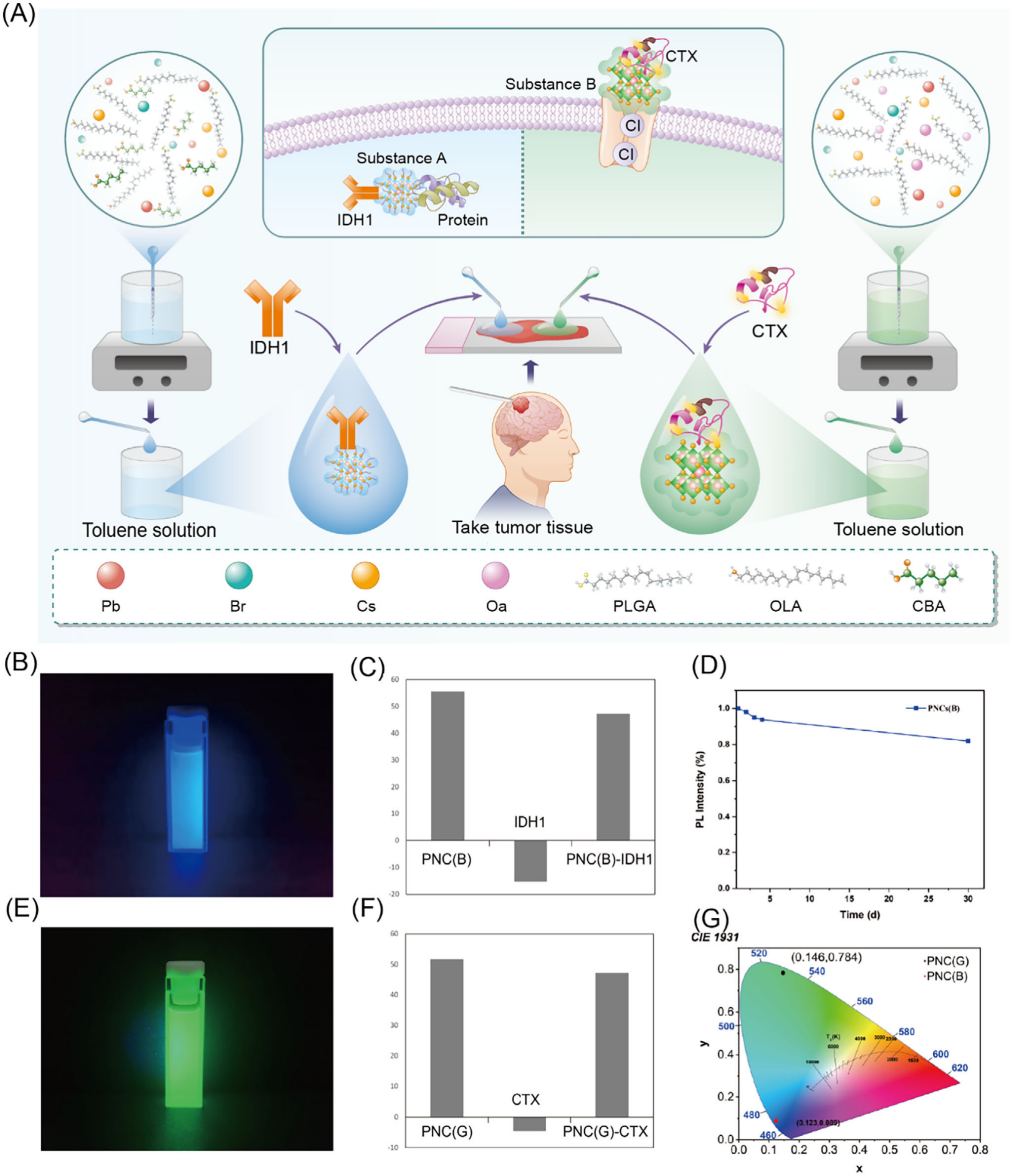
Synthesis of the blue and green dual‐probe. (A) Schematic diagram illustrating the preparation process of the blue and green quantum dot probes targeting CTX and IDH1‐mutant glioma, as well as the procedure for targeted staining of tissue samples; (B) luminescence image of the blue quantum dot probe; (C) zeta potential analysis of the blue quantum dot probe; (D) photoluminescence (PL) stability assessment of the blue quantum dot probe over 30 days; (E) luminescence image of the green quantum dot probe; (F) zeta potential analysis of the green quantum dot probe; (G) CIE coordinate diagram of the green quantum dots (PNCs (G)) and blue quantum dots (PNCs (B)).

To further verify the successful conjugation of antibodies onto PLGA‐coated perovskite quantum dots (PQDs), transmission electron microscopy (TEM) characterization was performed on the samples before and after antibody modification. As shown in Figure 4A, after PNCs (G) were conjugated with CTX antibody, the thickness of the formed protein layer was measured to be approximately 2 nm. The results in Figure 4B show that PNCs (B) exhibited a more distinct protein corona structure after conjugation with IDH1 antibody, with a protein layer thickness of about 5 nm. The obvious morphological changes and quantifiable differences in coating thickness directly confirm the successful antibody conjugation on both green and blue PQDs.

**FIGURE 4:**
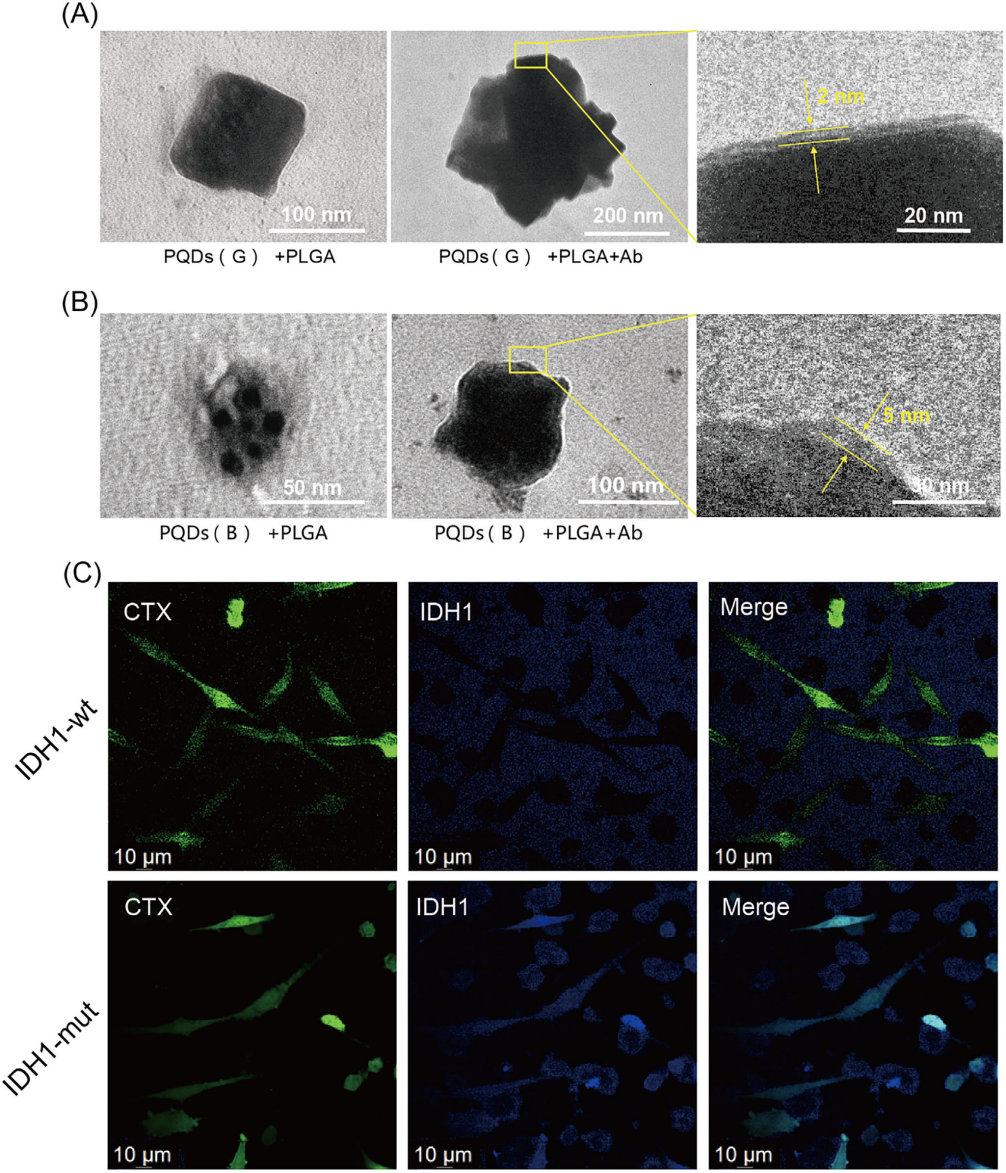
Specificity of antibody‐conjugated PLGA‐coated perovskite quantum dots. (A) Comparison of PNCs (G) before (left) and after (right) CTX antibody conjugation. A protein corona layer with a thickness of approximately 2 nm is formed on the surface of the probe after conjugation. (B) Comparison of PNCs (B) before (left) and after (right) IDH1 antibody conjugation. A protein corona layer of about 5 nm in thickness is observed on the probe surface following conjugation. (C) Targeting the specificity of the dual probes. Confocal images of U87 MG (upper) and IDH1‐U87 (lower) cells stained with PNC(G)‐CTX (green) and PNC(B)‐IDH1 (blue). Scale bar: 10 µm.

To verify the targeting specificity of the dual probes, confocal fluorescence imaging was conducted on U87 MG cells and IDH1‐U87 cells. The IDH1‐U87 cell line, which stably expresses the IDH1.R132H mutant protein, was purchased from the Cell Bank of the Shanghai Institute of Biochemistry and Cell Biology (Shanghai, China). As shown in Figure 4C, the PNC(G)‐CTX probe exhibited strong green fluorescence on the cell surface in both genotypes. In contrast, the PNC(B)‐IDH1 probe showed negligible blue fluorescence in U87 MG cells, but clear blue signals in the cytoplasm of IDH1‐U87 cells, confirming its high mutant‐specificity: it binds exclusively to the IDH1.R132H mutant protein without cross‐reactivity with endogenous wild‐type. Meanwhile, our previous studies have confirmed that the PNC(G)‐CTX probe can effectively distinguish glioma cells from normal brain cells [18]. Together, these results demonstrate that the dual probes we developed exhibit high targeting specificity.

### 2.4 Diagnostic Performance of Dual‐Color Probes

Using postoperative pathological diagnosis as the gold standard, we enrolled 56 intraoperative frozen section samples from patients and systematically evaluated the clinical diagnostic performance of the CTX probe and IDH1 probe. Core diagnostic indicators were quantified using 2×2 contingency table analysis, 95% confidence intervals calculated by the Wilson method, and one‐sided exact binomial testing. As shown in the contingency table for the CTX probe (Figure 5A), the sensitivity reached 100%, enabling 100% detection of all CTX‐positive glioma cases without missed diagnoses. The specificity was 85.7%, indicating a high rate of correct exclusion for CTX‐negative samples. The overall diagnostic accuracy was 98.2%, and the positive predictive value (PPV) and negative predictive value (NPV) were 98.0% and 100%, respectively, confirming the high reliability of the probe in identifying glioma boundaries. The contingency table for the IDH1 probe (Figure 5B) showed a sensitivity of 100%, allowing complete identification of all IDH1‐mutant positive cases without false negatives. The specificity was 87.1%, with a high correct exclusion rate for IDH1‐negative samples and no false positives. The overall diagnostic accuracy was 92.9%, and the PPV and NPV were 86.2% and 100%, respectively, demonstrating excellent diagnostic stability in IDH1 molecular subtyping. The correlation heatmap (Figure 5C) revealed a strong positive correlation between the CTX probe results and the CTX gold standard (r = 0.92), as well as between the IDH1 probe results and the IDH1 gold standard (r = 0.87). In contrast, the correlation between the two probes was low (r = 0.31), validating the rationality of probe design and confirming that the two probes can independently recognize their respective targets without significant cross‐interference. For further quantitative evaluation of the comprehensive diagnostic performance, five core indicators (sensitivity, specificity, accuracy, PPV, and NPV) were systematically analyzed (Figure 5D). The results demonstrated that both probes exhibited no obvious shortcomings in core diagnostic performance and fully meet the clinical requirements for rapid intraoperative diagnosis. Staining results in Figure 5E show that non‐glioma tissue exhibited only red nuclear fluorescence, whereas glioma cells displayed both red nuclei and green cytoplasmic signals. Additionally, IDH1‐mutant gliomas showed distinct blue fluorescence. With an overall accuracy of 91%, the dual‐color probe demonstrates considerable clinical potential for mutation detection in glioma.

**FIGURE 5:**
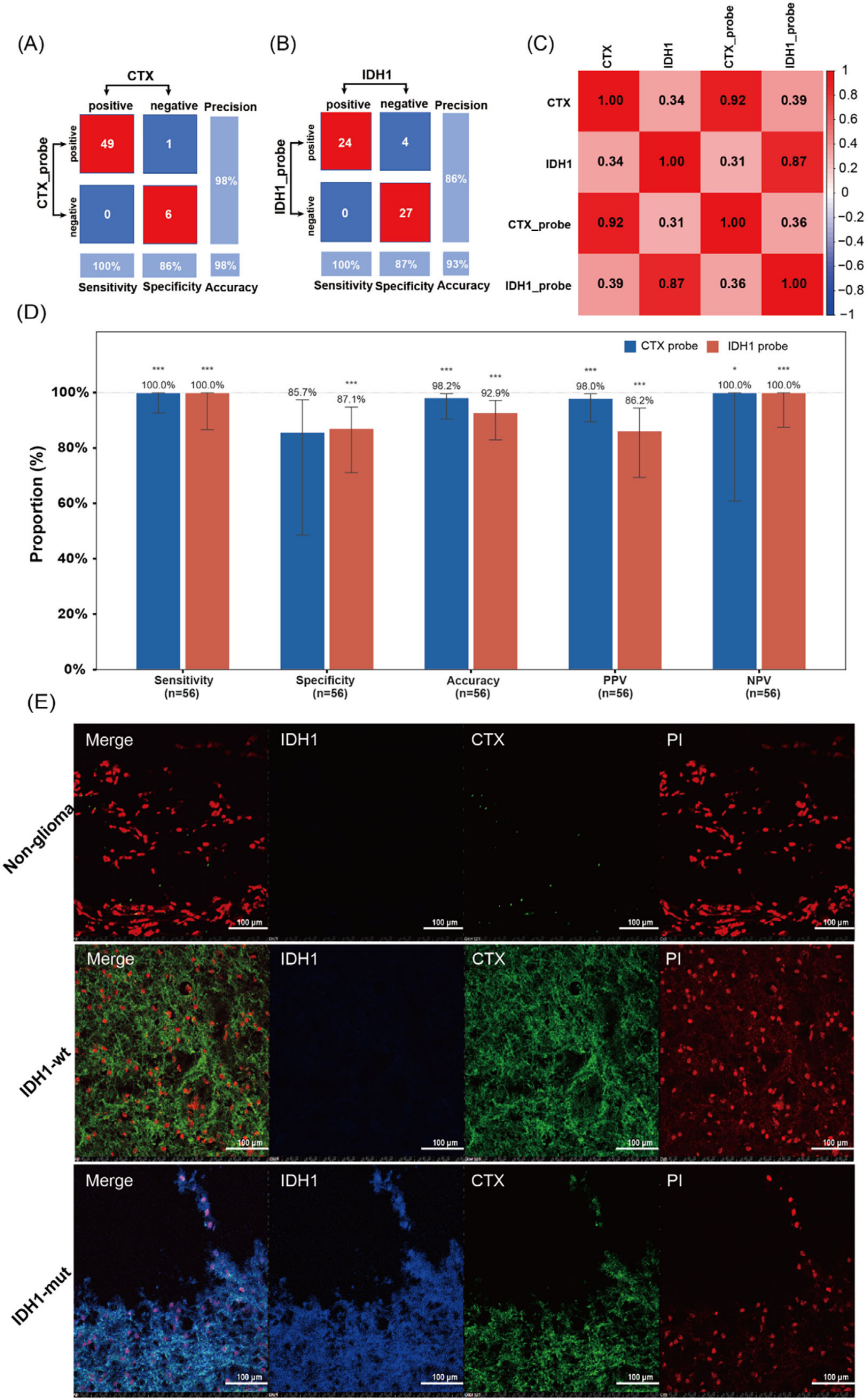
Diagnostic performance of the dual‐color probes. (A) Diagnostic performance of the CTX probe with pathological results as the gold standard; (B) diagnostic performance of the IDH1 probe with pathological results as the gold standard; (C) correlation heatmap analysis of the dual‐color probes; (D) comprehensive diagnostic performance metrics of the CTX and IDH1 probes. Error bars represent 95% confidence intervals calculated using the Wilson method. Asterisks indicate statistical significance (*p* < 0.05, *p < 0.01, **p < 0.001). (E) Staining images of the dual‐color probes. Red = PI nuclear staining, Green = CTX‐positive, Blue = IDH1‐mutant.

### 2.5 Personalized Precision Resection Strategy for Glioma Based on Dual‐Probe Detection Results

Based on the glioma cell detection results, this study proposes the glioma surgical resection strategy shown in Figure 6A. The CTX probe identifies tumor boundaries to guide supplementary resection, while the IDH1 probe simultaneously completes molecular subtyping. Finally, a tiered resection strategy is formulated based on the tumor location, enabling synchronous intraoperative decision‐making for both boundary recognition and molecular subtyping.

**FIGURE 6:**
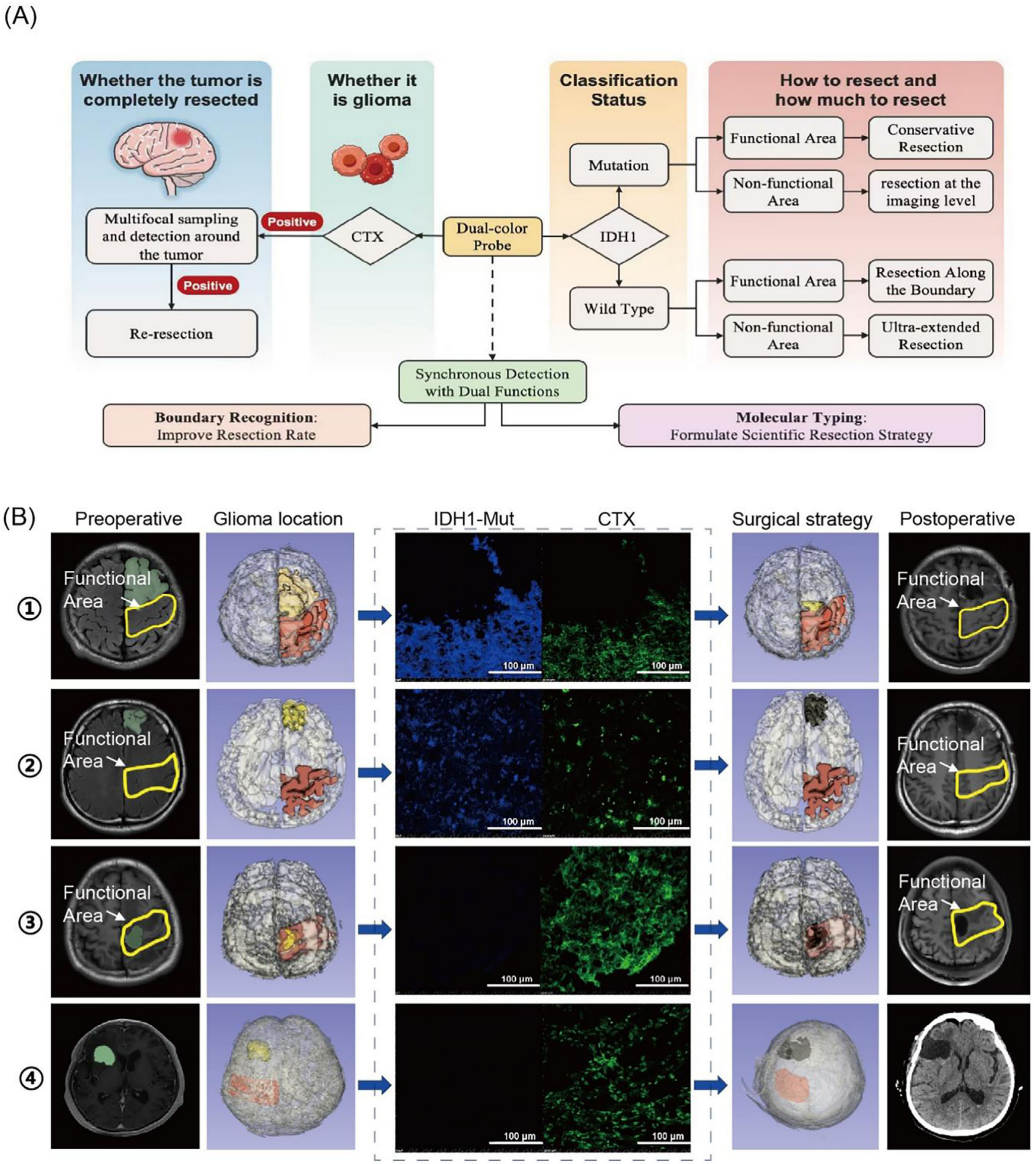
Framework of personalized precise resection strategy for gliomas and simulated clinical application scenarios based on dual‐probe detection results. (A) Decision tree of personalized, precise resection strategy for gliomas. (B) Simulated clinical application workflow of dual‐probe‐guided glioma resection. In MRI images, the tumor is labeled in green, and the boundaries of functional areas are marked with highlighted yellow wireframes. In 3D Slicer reconstructed images, the tumor entity is represented in yellow, and the functional areas are shown in red.

Figure 6B illustrates a simulated clinical application scenario for dual‐probe‐guided glioma resection constructed based on the validation findings of the present study (not actual surgical records). Both the yellow three‐dimensional tumor morphology and the red functional area structures in the figure were derived from preoperative multimodal magnetic resonance imaging (MRI) data of real clinical cases, with three‐dimensional reconstruction performed using the open‐source clinical neurosurgical software 3D Slicer to intuitively visualize the spatial anatomical relationship between tumor tissues and brain functional areas. Based on the perovskite quantum dot dual‐color probes, this study proposed a framework for precise glioma resection synchronously guided by tumor boundary identification combined with IDH1 molecular subtyping. Figure 6B visually demonstrates the implementation workflow and translational value of this strategy in future clinical operations, providing methodological support for subsequent prospective clinical trials.

## 3 Discussion

For the neuropathological diagnosis of brain gliomas, the fifth edition of the World Health Organization Classification of Tumors of the Central Nervous System (2021) explicitly includes several molecular markers for the integrated pathological diagnosis of gliomas. Among these molecular markers, the mutation status of IDH1 is particularly important for treating glioma patients [19]. This study also further confirmed, based on bioinformatics analysis results, that glioma patients with IDH1 mutations have better survival outcomes and chemosensitivity than patients with wild‐type IDH gliomas, and the difference in survival benefits is related to the extent of maximal tumor resection [20, 21]. Therefore, when the tumor is located near functional areas, formulating scientific surgical resection strategies based on IDH1 mutation status is of great significance. However, due to the invasive growth of glioma tissue into normal brain tissue, the tumor boundary is often unclear and cannot be fully determined by the naked eye. At present, conventional clinical diagnostic methods either take a long time or have certain limitations: for example, although methods such as intraoperative neuronavigation, magnetic resonance imaging (MRI), and ultrasonography can identify tumor boundaries through imaging, they cannot provide histopathological information [22]; traditional microscopic examination of intraoperative frozen tissue sections is usually only used for preliminary diagnosis and cannot provide molecular or genetic‐level information; similarly, although cytopathological testing can determine glioma grading based on tumor morphology, it cannot accurately diagnose IDH mutation status, limiting its application in intraoperative real‐time detection [23]; immunohistochemical staining involves cumbersome procedures, and antibody quality and antigen retrieval effectiveness significantly affect the final results. It is not only time‐consuming to operate but also requires operators to have high technical proficiency, making it difficult to meet the needs of intraoperative real‐time detection [24].

Fluorescent probe technology is the core method for solving intraoperative rapid molecular diagnosis. Among them, traditional organic fluorescent dyes (such as FITC, Cy3, and Alexa Fluor series) are currently the most commonly used labeling materials. However, limited by their inherent defects in optical properties, it is difficult to achieve simultaneous and rapid detection of multiple targets. In contrast, perovskite quantum dots (PQDs), as a new generation of fluorescent labeling materials, exhibit significant technical advantages in intraoperative pathological diagnosis scenarios: first, ultra‐high fluorescence quantum yield. PLGA‐coated CsPbX_3_ PQDs have been widely reported to possess a high fluorescence quantum yield (>90%), which is significantly higher than that of traditional organic dyes such as FITC and Cy3 [25]. This enables the probes to generate strong fluorescent signals at low concentrations, which not only reduces the dosage of probes but also can clearly identify a small number of positive cells in the tumor microinfiltration area. It is crucial for accurately delineating the tumor boundary during surgery. What's more, the extremely narrow emission peak enables crosstalk‐free dual‐color imaging. In this study, the full width at half maximum (FWHM) of the emission peak of blue PQDs is 17 nm, and that of green PQDs is 19 nm, which is much narrower than the 50–100 nm of organic dyes. It solves the serious spectral overlap problem in multi‐color imaging of traditional organic dyes [26], ensuring that the signals of the two channels (CTX in green and IDH1 in blue) are completely independent without complex spectral separation, thus guaranteeing the accuracy of diagnostic results. Verification in this study shows that the fluorescence intensity of PQD probes decreases by < 5% within 30 days at room temperature in the dark, and by <10% after 30 min of continuous laser irradiation. In contrast, traditional organic dyes undergo rapid photobleaching under the same conditions [27], which cannot meet the needs of multiple observations and repeated imaging during surgery. The direct labeling probes based on PQDs do not require cumbersome steps such as antigen retrieval and secondary antibody incubation, with a total staining time of only 10 min. Combined with frozen section preparation, the entire detection can be completed within 30 min. In contrast, intraoperative rapid immunohistochemistry based on organic dyes requires at least 1–2 h, which cannot meet the time window requirements for real‐time intraoperative decision‐making. Objectively, the biosafety of lead‐based PQDs is the core concern for clinical translation. Up to now, this probe is only used to stain in vitro frozen tissue sections, instead of entering the patient's body, which has no risk of systemic exposure. At the same time, this study adopts a PLGA polymer coating strategy, forming a dense shell through the coordination between carboxyl groups and Pb^2^^+^, which can effectively reduce the risk of lead leakage.

The results confirmed that the dual‐color probe exhibited excellent diagnostic performance in clinical frozen sections. The green probe targeting chlorotoxin (CTX) specifically recognizes chloride channel proteins on glioma cell membranes with 100% sensitivity and 98% accuracy, enabling precise discrimination between glioma and non‐glioma tissues and effective identification of micro‐invasive tumor borders undetectable by the naked eye. The blue probe targeting IDH1 mutation showed 100% specificity and 93% accuracy, allowing reliable determination of IDH1 mutation status in glioma cells at the cytoplasmic level. The overall accuracy of combined detection using the two probes reached 91%.

This probe enables the distinction of tumor boundaries from normal brain tissue at the histopathological level, thereby improving the precision of tumor resection. It does not require clinicians to possess advanced operational skills, reduces procedural complexity and detection costs, and shortens the turnaround time for molecular pathological results. Molecular diagnostic information of tumor tissues can be obtained within 30 min, significantly improving the efficiency of intraoperative pathological detection. Surgeons can optimize intraoperative strategies based on this information to achieve precise and personalized resection of gliomas.

Based on the dual‐color perovskite quantum dot probes, this study proposes a precision glioma resection framework guided by simultaneous tumor boundary identification and IDH1 molecular subtyping. The standardized preclinical resection strategies under this framework are summarized as follows: ①For IDH1‐mutant gliomas located in functional areas, conservative resection is prioritized to preserve neurological function; postoperative brain imaging is adopted to verify the safe distance between resection margins and functional areas. ②For IDH1‐mutant gliomas in non‐functional areas, maximal safe resection is performed to achieve thorough tumor removal as far as possible. ③For IDH1 wild‐type gliomas within functional areas, resection along tumor boundaries is conducted to minimize neurological damage. ④For IDH1 wild‐type gliomas in non‐functional areas, extended resection beyond the visible tumor boundary is implemented to optimize therapeutic outcomes.

The complete preclinical implementation workflow corresponding to the above strategies is described as follows: ①Preoperative stage: Multimodal MRI data of patients are collected, three‐dimensional models of tumors and adjacent critical functional areas are reconstructed via 3D Slicer, and the initial surgical resection plan is formulated in accordance with the Chinese Guidelines for Integrated Diagnosis and Treatment of Gliomas. ②Intraoperative stage: Tissue specimens from the tumor core and safe adjacent regions of functional areas are harvested for frozen section preparation. Staining is completed within 30 min using the dual‐color probes developed in this study, enabling synchronous acquisition of tumor boundary information and IDH1 mutation status. Real‐time intraoperative adjustment of the surgical resection protocol is performed based on the detection results. ③Postoperative validation stage: Probe detection outcomes are compared with the gold standards of conventional postoperative pathological examination and genetic testing. Meanwhile, postoperative MRI re‐examination is performed to compare the actual resection scope with the probe‐guided planned resection scope, so as to systematically validate the clinical efficacy of the proposed strategy.

The above preclinical workflow indicates that rapid intraoperative identification of tumor boundaries and IDH1 mutation status can provide critical molecular pathological evidence for neurosurgeons to formulate more precise glioma resection strategies. The dual‐target and dual‐color probes developed in this study enable rapid intraoperative ex vivo pathological diagnosis with simultaneous visualization of tumor cells and IDH1 mutation status. The total detection duration is comparable to that of traditional intraoperative frozen hematoxylin‐eosin (HE) staining, allowing seamless integration into existing clinical surgical workflows. This novel technical approach enables surgeons to comply with clinical guidelines [28] and achieve optimized individualized glioma resection, thereby exhibiting significant potential for clinical translation.

## 4 Conclusions

In this study, green and blue perovskite quantum dot (PQD) aqueous solutions were successfully synthesized by introducing distinct ligands into the precursor solution. The PLGA‐coated blue and green PQDs exhibited similar particle sizes (56–58 nm) and excellent aqueous stability. Through a charge‐coupled conjugation strategy, green quantum dots (PNCs (G)) and blue quantum dots (PNCs (B)) were respectively conjugated with IDH1 antibodies and CTX antibodies, resulting in the development of a dual‐color, dual‐target PQD‐based probe. This probe enables rapid intraoperative in vitro pathological diagnosis by simultaneously identifying glioma cells and determining IDH1 mutation status. It holds significant potential for practical application in achieving precise resection of gliomas located near functional brain areas.

## 5 Experimental Section/Methods

### 5.1 Reagents

The following reagents were used in this study. Unpurified CsBr, PbBr2 (Xi'an Polymer Light Technology Corp.), carboxyl‐terminated polylactide‐co‐glycolide (OH‐PLGA‐COOH, Beijing Cowin Bioscience Co., Ltd., MW: 110,000), oleylamine (OAm; J&K), oleic acid (OA), chlorobutyric acid (CBA), N,N‐dimethylformamide (DMF) (Aladdin), toluene (Sinopharm), and chlorotoxin peptide (CTX) were purchased from Shanghai Yuanye Biotech Co., Ltd. The IDH1‐R132H clone H09 was obtained from Dianova (Hamburg, Germany). Ultrapure water was provided by a Millipore Milli‐Q system (18 MΩ·cm).

### 5.2 Patient Data Collection

All tissue specimens used in this study were obtained from patients who underwent surgical treatment in the Department of Neurosurgery at the First Medical Center of the Chinese PLA General Hospital. A total of 49 tumor core tissue sections from glioma patients were collected. The inclusion criteria were as follows: (A) patients diagnosed with glioma by preoperative imaging and postoperative pathological examination; (B) patients with confirmed IDH1 mutation status via postoperative molecular testing; and (C) patients for whom the required intraoperative frozen tumor sections were preserved. Additionally, tissue sections from the core regions of 7 non‐glioma tumor patients were included. The study was approved by the local ethics committee (Ethics Approval No.: S2021‐610‐01), and written informed consent was obtained from all patients prior to surgery.

### 5.3 Synthesis of Nanocrystals

PNCs(G) and PNCs(B) were synthesized as follows. First, 0.2 mmol of CsBr, 0.2 mmol of PbBr2, and 50 mg of OH‐PLGA‐COOH were dissolved in 5 mL of DMF and stirred at 60°C for 30 min to ensure complete dissolution. For PNCs(G) synthesis, 0.5 mL of OA and 0.25 mL of oleamine were added to the precursor solution for stabilization. For PNCs(B) synthesis, 1 mmol of CBA and 1 mmol of oleamine were added to the precursor solution. Then, 0.75 mL of the resulting solution was slowly added to 15 mL of a toluene solution and vigorously stirred at 60°C to obtain P‐PNCs in toluene solution. Subsequently, the solution was vigorously stirred and heated at 45°C for 6 h to react fully. The resulting solution was centrifuged at 8500 rpm for 10 min to recover the precipitate. After drying in a fume hood, P‐PNC powders were obtained and collected for subsequent use.

### 5.4 Synthesis of Nanocrystals and Probes

To synthesize the probes, PNCs(G) and CTX polypeptides or PNCs(B) and IDH1 antibodies were coupled via a self‐assembly mechanism based on electrostatic adsorption. In this process, 0.1 g of each P‐PNC powder was dissolved in ultrapure water (1 mL) at room temperature. Then, 100 µL of CTX polypeptide (1 mg mL^−1^) was added to the PNCs(G) solution, and 100 µL of IDH1 antibody (1 mg mL^−1^) was added to the PNCs(B) solution for 30 s. The probes were then stored at 4°C.

### 5.5 Material Characterization

X‐ray diffraction (XRD) patterns of PNCs(G) and PNCs(B) powders were collected using an X‐ray diffractometer (Rigaku miniflex600) with monochromatic Cu‐Kα radiation (λ = 1.5418 A). Tests were performed at 40 kV and 15 mA, over an angular range of 10°–80° (2θ), with a scan rate of 5°/min and a step size of 0.02°. Fourier transform infrared (FTIR) spectra for PNCs(G) and PNCs(B) were obtained using an FTIR spectrometer (Bruker Tensor 2) in attenuated total reflectance mode. The photoluminescence (PL) and time‐resolved PL spectra for PNCs(G) and PNCs(B) were obtained using a fluorescence spectrometer (FLS 980, Edinburgh Instruments Ltd.) at an excitation wavelength of 365 nm. Scanning electron microscopy (SEM) images of PNCs(G) and PNCs(B) were obtained using a field‐effect SEM system. Transmission electron microscopy (TEM) images of PNCs(G) and PNCs(B) were obtained using a field‐emission TEM system (FEI Talos200X, 200 kV). X‐ray photoelectron spectroscopy spectra were recorded using an AXIS Ultra DLD surface analysis system (Kratos Analytical) under ultra‐vacuum.

### 5.6 Cell Imaging

U87 MG cells and U87 cells stably carrying the IDH1.R132H mutation were purchased from the Cell Bank of Shanghai Institute of Biochemistry and Cell Biology, Chinese Academy of Sciences. This cell line is an isogenic glioma cell model derived from U87 MG, which specifically harbors the IDH1 R132H mutation [29]. Cells were cultured in Minimum Essential Medium (MEM) supplemented with 10% fetal bovine serum, 1% penicillin‐streptomycin solution, 1.5 g L^−1^ sodium bicarbonate and 0.11 g L^−1^ sodium pyruvate. All cells were cultured in Corning 430167 culture dishes at 37°C in a humidified incubator with 5% CO_2_. The culture medium was refreshed every 48 h, and cell subculture was performed when cell confluence reached 80%–90%.Cells were fixed with 2 mL 4% paraformaldehyde for 15 min and rinsed three times with phosphate buffered saline (PBS). Subsequently, 2 mL goat serum blocking solution was added to block unreacted active sites for 30 min at room temperature, followed by another three PBS washes. PNCs(B) and PNCs(G) probes at a concentration of 100 µg mL^−1^ were added to the cells with a volume of 500 µL, and incubated for 5 min respectively at room temperature. After three thorough rinses with PBS, cellular fluorescence images were captured using a Leica confocal microscope, and subsequent image processing.

### 5.7 Staining of Frozen Tissue Sections

Frozen sections of tissue specimens were fixed with 4% paraformaldehyde and rinsed with phosphate‐buffered saline (PBS). To block unreacted active sites, the specimens were soaked in a goat serum blocking solution (2 mL) for 10 min and then cleaned with PBS three times. Propidium iodide was added to the frozen tissue sections and incubated in the dark for 5 min. Then, the sample was washed with PBS and rinsed with ultrapure water. The slices were subsequently incubated with the PNCs(G)‐CTX probe in the dark for 5 min and washed with ultrapure water in a dyeing box for 1 min. The slices were then incubated with the PNCs(B)‐IDH1 probe in the dark for 5 min and washed with ultrapure water in a dyeing box for 4 min. After washing, imaging was performed using a Nikon C2 confocal microscope at excitation wavelengths of 405, 488, and 561 nm [17] Supproting informaton.

## Funding

This work was financially supported by the National Nature Science Foundation of China (Grant 52473271) and the National Key R&D Program of China (Grant No. 2024YFB3815200).

## Ethical Statement

This study was approved by the institutional review board of the Chinese PLA General Hospital (S2021‐610‐01), and was done in compliance with the World Medical Association Declaration of Helsinki.

## Conflicts of Interest

The author(s) declared no potential conflicts of interest with respect to the research, authorship, and/or publication of this article.

## Supporting information




**Supporting File**: advs76379‐sup‐0001‐SuppMat.docx.

## Data Availability

Any additional information required to reanalyze the data reported in this paper is available from the lead contact upon request.
