# Supplementary material for: Rapid In Vitro Pathological Diagnosis of Glioma Using Dual‐Color Quantum Dot Probes for Precise Intraoperative Resection
Source: Adv Sci (Weinh). 2026 Jul 6:e76379. Online ahead of print. doi: 10.1002/advs.76379 (PMC13335713; doi:10.1002/advs.76379)
Supplement: Supplementary file 1 — Supporting File: advs76379‐sup‐0001‐SuppMat.docx. [file ADVS-9999-e76379-s001.docx]

**Supporting Information**

**Rapid In Vitro Pathological Diagnosis of Glioma Using Dual-Color Quantum Dot Probes for Precise Intraoperative Resection**

*Dongdong Wu#, Chenxuan Yang#, Yuanbin Wu#, Minghang Liu, Yizhi Zhang, Yuang Cai, Yibo Zhang,Jimei Chi, Hengchao Ma, Ding Zhang, Chenqing Liu*, Meng Su*, Guochen Sun**

#These authors contributed equally to the manuscript.


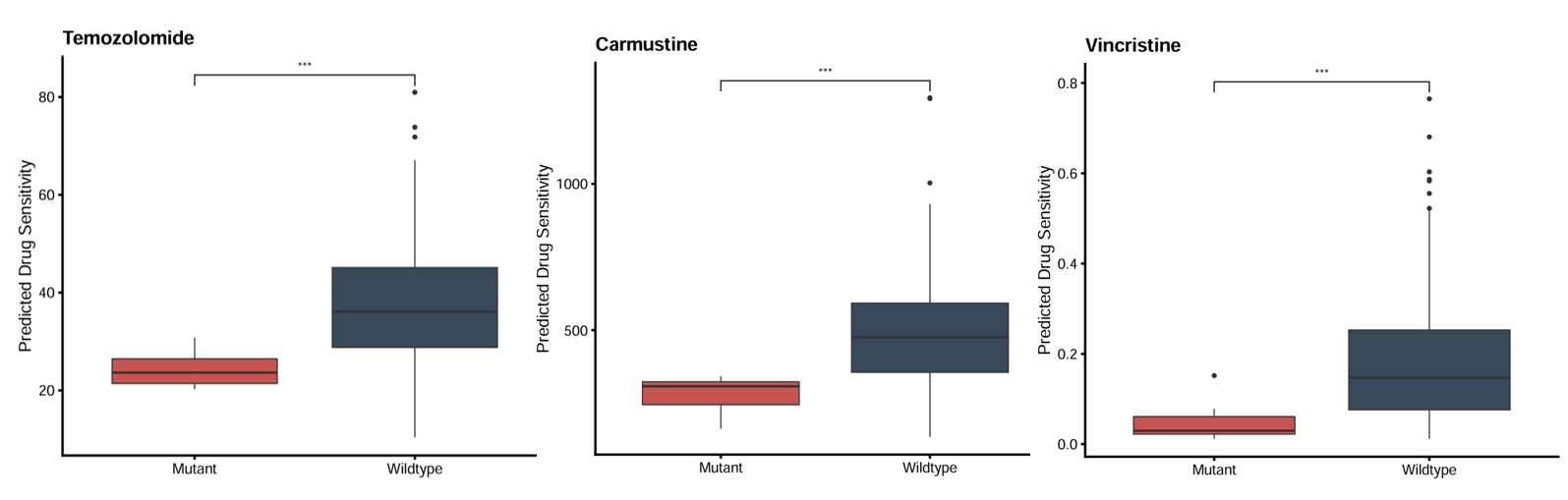


**S1.** OncoPredict was used to predict the sensitivity of Vincristine (VCR) in glioma patients. It was found that IDH1-mutant glioma patients showed higher drug sensitivity to these three drugs, and their IC50 values were significantly lower than those of wild-type patients (p < 0.05).


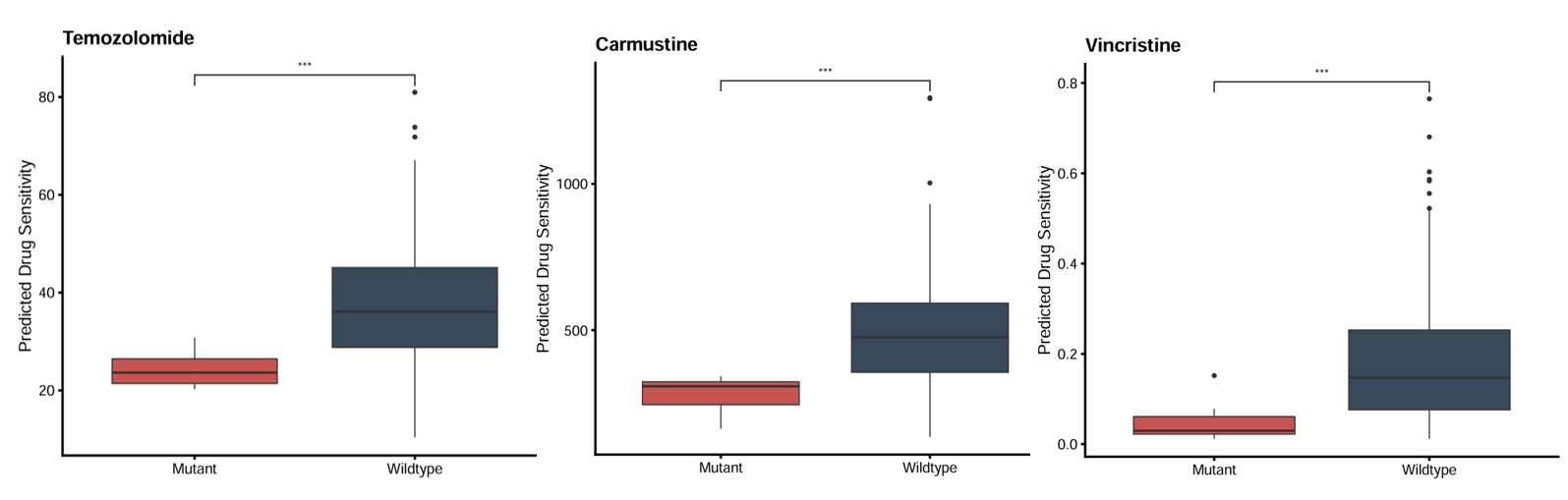


**S2.** OncoPredict was used to predict the sensitivity of three common chemotherapeutic drugs, including Carmustine (BCNU), in glioma patients. It was found that IDH1-mutant glioma patients showed higher drug sensitivity to these three drugs, and their IC50 values were significantly lower than those of wild-type patients (p < 0.05).


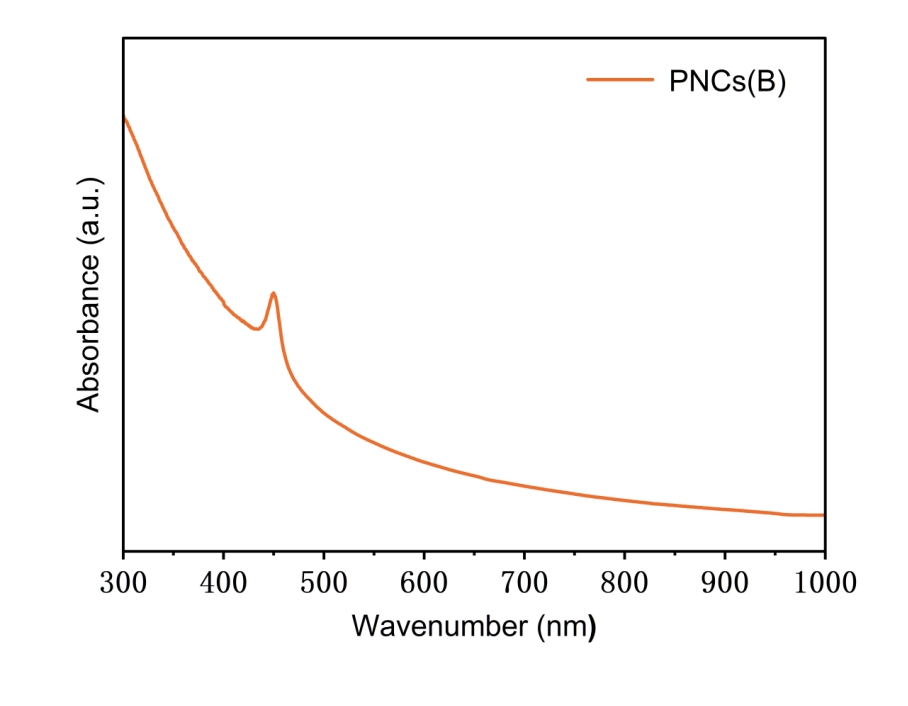


**S3.** The UV spectrum of PNC(B) exhibits a broad exciton absorption band and a sharp exciton absorption peak at 450 nm.


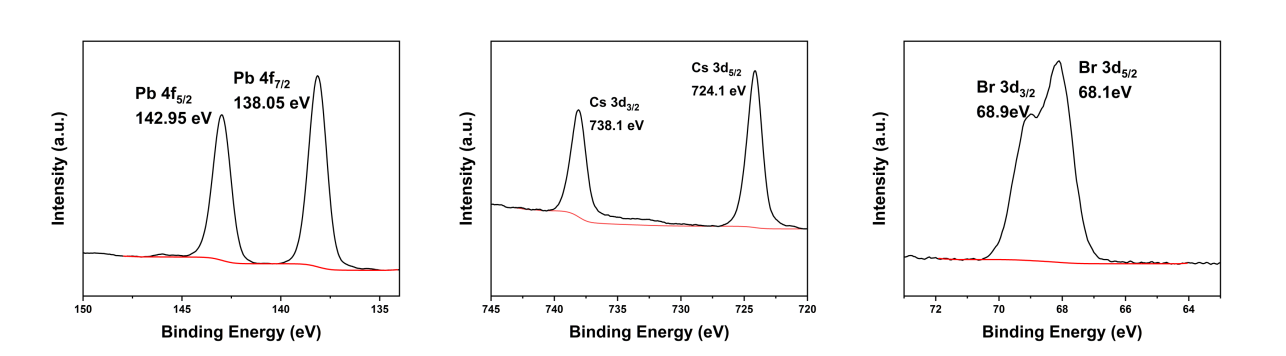


**S4.** XPS analysis of PNC(G) corresponding to (B) Pb 4f, Cs 3d, and (C) Br 3d.


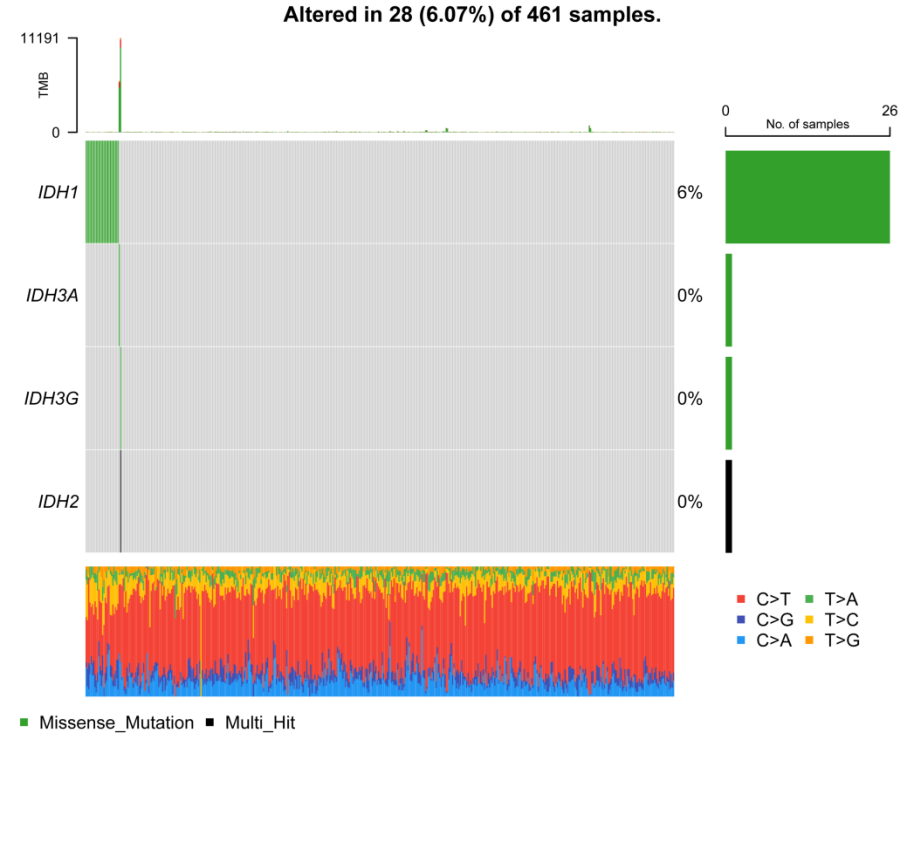


**S5. Mutation Characteristics of the IDH Gene Family**

The mutation landscape of the IDH gene family (IDH1, IDH2, IDH3A, IDH3G) in tumor samples is shown, with IDH1 exhibiting the highest mutation rate (6%), primarily as missense mutations, and correlating with an increased tumor mutation burden (TMB). The mutation spectrum at the bottom indicates that C>T transitions are the predominant mutation type in IDH1, suggesting a potential mutation mechanism.


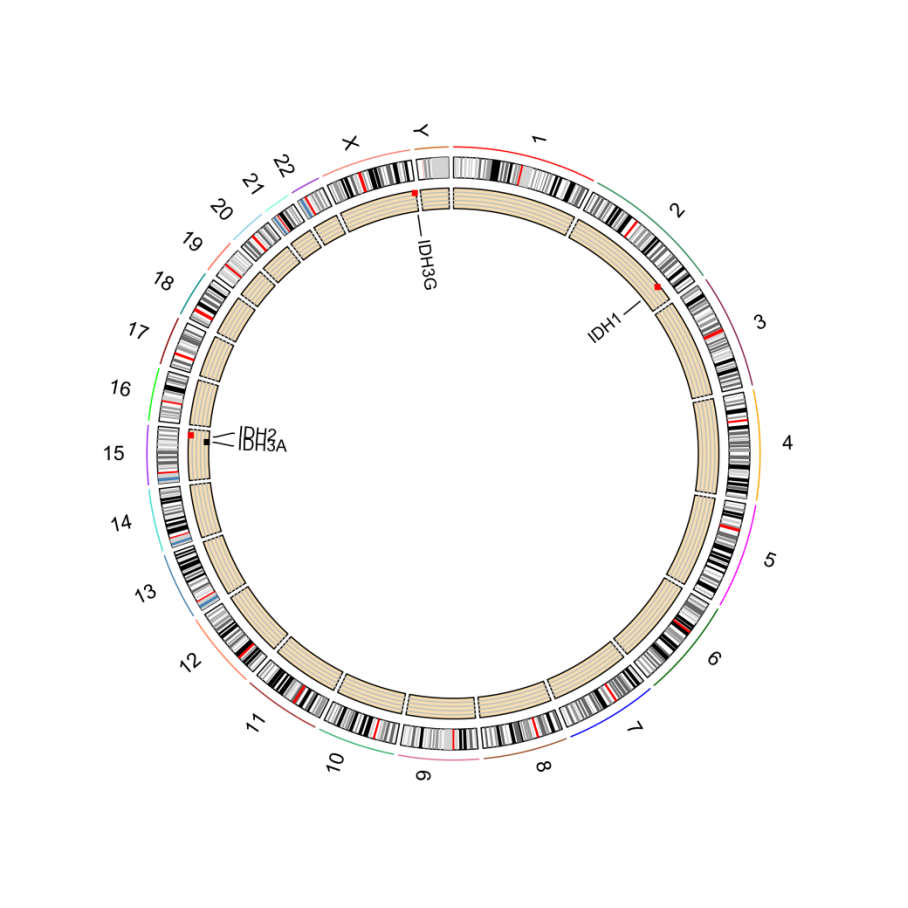


**S6. Copy Number Variation (CNV) Circos Plot**

Genome-wide CNV alterations are displayed, with IDH1, IDH3A, and other genes labeled. Red markings indicate regions with a higher frequency of copy number amplification, while black markings represent regions with a higher frequency of copy number deletion. CNV in the IDH1 region on chromosome 2 suggests that genetic alterations in this locus may play a crucial role in tumor progression and prognosis.
